# Reliability of using an ultrasound scoring measure for juvenile localized scleroderma (jLS)

**DOI:** 10.1186/1546-0096-10-S1-A69

**Published:** 2012-07-13

**Authors:** Suzanne C Li, Melissa S Liebling, Molly Dempsey-Robertson, Andrea S Doria, Stephanie Edgerton, Carsten Hamer, Jose Jarrin, Tanicka Kornyat, Michael Malone, Arun Mohanta, Sven Opitz, Faridali Ramji, Shuzhen Zhang, Knut Wittkowski

**Affiliations:** 1 Hackensack University Medical Center, Hackensack, NJ, USA; 2 Hospital for Sick Children, Toronto, ON, Canada; 3 Rockefeller University, New York City, NY, USA; 4 Schön Klinik Hamburg Eilbek, Hamburg, Germany; 5 Texas Scottish Rite, Dallas, TX, USA; 6 University of Oklahoma, OKC, OK, USA

## Purpose

Although ultrasound shows great potential for aiding assessment of LS disease activity, its use has been limited because both image acquisition and interpretation are operator dependent. To facilitate evaluating and to standardize interpretation of jLS ultrasound scans, we generated a scoring measure for evaluating echogenicity and vascularity differences (U-DA, Ped Rheum 2010;8:14). Objective: To assess the reliability of an ultrasound scoring measure (U-DA) for jLS.

## Methods

A meeting held from 2/21 to 2/24/09 brought together 12 radiologists and sonographers from 5 institutions for hands-on training in acquiring and scoring ultrasound scans of jLS patients. The group had initially met in 2007, and had developed a preliminary ultrasound scoring measure. This preliminary measure was reviewed in conjunction with scans showing the range of sonographic differences that had been observed to date in jLS; this review led to an expansion of scoring ranges. Following finalization of the U-DA, the group was led in the scoring of a jLS scan by Dr. Liebling, and then attendees independently scored two other scans. These scores were jointly reviewed to ensure that participants were comfortable with applying the U-DA measure. Participants then independently scored a set of 16 jLS scans; each participant had a different order of the scans. Ten of the 11 scorers scored these scans a second time on a different day, with scans presented in a different order from the original set. Intraclass correlation scores for total echogenicity and vascularity were calculated to determine intra-rater reliability. The median score for each of the 6 U-DA parameters (echogenicity and vascularity separately scored for the dermis, hypodermis, and deep tissue layer) was determined, and the distribution of each rater’s score relative to the median plotted.

## Results

All raters showed moderate to high intra-rater reliability for scoring total echogenicity (ICC 0.591 to 0.806). Poorer intra-rater reliability was found for scoring total vascularity, ranging from poor to high agreement. When evaluating the median and range of scores for each U-DA parameter, for most scans, most raters scored at or ± 1 within the median. However, for a few scans, some parameters showed no consensus or a wide range of scores. Deep tissue layer parameters were more likely to be problematic than those for other layers.

## Conclusion

Good intra-rater agreement was found for scoring total echogenicity. There was variable intra-rater reliability for scoring total vascularity, and inter-rater reliability varied among the study scans, with some U-DA parameters showing good agreement and others widely divergent scores. Scoring was more often problematic for the deep tissue layer. More review of the scans showing poorer inter-rater agreement, and more focus on deep tissue layer evaluation, may lead to improved inter-rater scoring reliability.

## Disclosure

Suzanne C. Li: Arthritis Foundation, 2; Melissa S. Liebling: None; Molly Dempsey-Robertson: None; Andrea S. Doria: None; Stephanie Edgerton: None; Carsten Hamer: None; Jose Jarrin: None; Tanicka Kornyat: None; Michael Malone: None; Arun Mohanta: None; Sven Opitz: None; Faridali Ramji: None; Shuzhen Zhang: None; Knut Wittkowski: NIH, 2.

